# A Central Medical School for New York

**Published:** 1911-01-14

**Authors:** 


### COLUMBIA UNIVERSITY AND THE PRESBYTERIAN HOSPITAL.

### A CENTRAL MEDICAL SCHOOL FOR NEW YORK.

(From a Special Correspondent.)

Among the hospitals of New York City none, perhaps, is better known or enjoys a more established reputation than the fine Presbyterian Hospital, even although it cannot, like the New York Hospital and a few other competitors, look back upon more than a century of work. Founded in 1868, it has made its reputation through merit. It may claim, with justice, to rank among the best managed institutions in the city, to possess a staff which is second to none, so far as professional standing is concerned, in America, and to have organised an excellent nursing service and established one of the best nursing schools in the sub-continent. It is therefore not to be wondered at that those responsible for the scheme of organising a large central medical school for New York should have decided to secure the co-operation and support of the authorities of this hospital. The scheme whereby the city of New York is to obtain a magnificent new hospital and a central medical school that may deservedly be styled a "university" has only recently been completed, and we are now able to give further details with regard to it.

### Rebuilding the Presbyterian.

In 1908 the authorities of the Presbyterian Hospital decided to embark on an extensive rebuilding scheme. The Presbyterian, although not, comparatively speaking, an old hospital, cannot, from an architectural and construction point of view, honestly be considered as an up-to-date institution, and the management therefore resolved to spare no pains to bring it into line with the latest improvements in hospital design.

In that year Mr. J. S. Kennedy, President of the Hospital, celebrated the fiftieth anniversary of his wedding by presenting the hospital with a million dollars, on condition that the money should be spent on a new administration building. After discussion with the medical staff, it was decided to acquire a new site, and so build and equip a new hospital according to the latest models, and when, in 1909, Mr. Kennedy died, he left an additional two million and a half dollars for this purpose. A committee, among whom was Professor James, of Columbia University, who is a member of the hospital medical staff, was appointed to consider the whole question of extension very carefully. Dr. James visited Europe in 1909, and inspected various model hospitals, while the other members of the committee busied themselves in the same direction in America. The plans, ultimately approved by the board of management's building committee, of which Mr. Robert W. De Forest is the Chairman, provide for the erection of an entirely new hospital, which will be one of the finest in the world, and promises to be the finest in America. A site has been secured on elevated open ground in the neighbourhood of the Rockefeller Institute and Flower Hospital, the central hospital building being planned to occupy the entire block between 67 and 68 Streets and Avenue A and the East River.

### The Alliance with Columbia University.

When Mr. De Forest became Chairman of the Building Committee it was felt by the hospital authorities that the time was opportune to ally the institution with a good medical school. The Education Committee of the Columbia University had already tentatively approached the Board with a proposal to co-operate in such a scheme, and this was favourably entertained, after the authorities had assured themselves that the interests of the hospital, which they rightly desired should be paramount in any case, would be materially helped instead of hindered by such an alliance. The question of money was an important one. The Board of the Presbyterian Hospital, acting on the principle which has been so generally followed in London, made it a condition of the agreement between the hospital and the university that the money necessary to defray expenses for research work and for purely collegiate purposes should come out of an entirely independent fund specially contributed for this particular purpose—in other words, that no portion of the funds of the hospital, given for the relief of the sick, should be spent upon research or medical teaching. It is gratifying to be able to record that this condition was at once accepted by the Columbia University. Mr. Edward S. Harkness, Chairman of the Executive Committee of the Central Council of Charities Organisation, thereupon announced that he and another friend of the hospital, and of the university were willing to contribute immediately a sum of nearly a million and a half dollars to be specially earmarked for educational purposes, and a further sum of 300,000 dollars for the erection of a special surgical ward.

### The Question of Funds.

The financial difficulties having thus been settled in an exemplary fashion, the formal agreement between the hospital and the university has just been concluded. By it the hospital, with the approval of its trustees, undertakes to enter into an alliance with the university, and to allow medical teaching to be conducted in its wards. The members of the staff of the Presbyterian will henceforward be professors of the medical faculty in the university, or adjunct professors, as the case may be, and no new appointments will be made without the sanction of the university. For all practical purposes, therefore, the Presbyterian becomes a university hospital, and may be likened to the Glasgow or Edinburgh Infirmary or to the University College Hospital, or King's. The mutual benefits resulting from this co-operative alliance are easy to estimate in one way; difficult, because less tangible, in another. As Mr. De Forest ably expressed it in an article in the *New York Times*, "The fundamental principle of the alliance, calling upon the university to meet the expenses of scientific investigation, means that both institutions will become more important factors in the cause of preventive medicine, and that the hospital will be relieved of the increasing expense incident to the modern development of medicine and surgery, such as its pathological, bacteriological, and *x*-ray departments." The alliance has been very well received in New York, and institutional workers and professional men in this country will join with us in heartily congratulating both the Presbyterian Hospital and Columbia University upon the carrying out of an arrangement which bids fair to be so eminently advantageous to both.

